# Phase II open-label study of recombinant circularly permuted TRAIL as a single-agent treatment for relapsed or refractory multiple myeloma

**DOI:** 10.1186/s40880-016-0140-0

**Published:** 2016-09-08

**Authors:** Yun Leng, Lugui Qiu, Jian Hou, Yaozhong Zhao, Xuejun Zhang, Shifang Yang, Hao Xi, Zhongxia Huang, Ling Pan, Wenming Chen

**Affiliations:** 1 Department of Hematology, Beijing Chao-Yang Hospital, Capital Medical University, 8 Gongren Tiyuchang Nanlu, Chaoyang District, Beijing, 100020 P. R. China; 2 Department of Lymphoma Center, Institute of Hematology and Blood Diseases Hospital, Chinese Academy of Medical Science & Peking Union Medical College, Tianjin, 300020 P. R. China; 3 Department of Hematology, Shanghai Changzheng Hospital, The Second Military Medical University, Shanghai, 200003 P. R. China; 4 Department of Hematology, The Second Hospital of Hebei Medical University, Shijiazhuang, 050000 Hebei P. R. China; 5 Beijing Sunbio Biotech Co., Ltd., Beijing, 100176 P. R. China; 6 Department of Hematology, West China Hospital, Sichuan University, Chengdu, 610041 Sichuan P. R. China

**Keywords:** Recombinant human circularly permuted TRAIL, Phase II trial, Relapsed or refractory multiple myeloma, Efficacy, Safety

## Abstract

**Background:**

Despite the recent development of new therapies, multiple myeloma (MM) remains an incurable disease. Thus, new, effective treatments are urgently needed, particularly for relapsed or refractory MM (RRMM). In an earlier phase I study, a novel form of recombinant human Apo2L/tumor necrosis factor-related apoptosis-inducing ligand (TRAIL) that is currently in clinical development for the treatment of hematologic malignancies, i.e., circularly permuted TRAIL (CPT), was well tolerated at a dose of 2.5 mg/kg per day and showed promising preliminary activity in patients with RRMM. This phase II, open-label, multicenter study further investigated the efficacy and safety of 2.5-mg/kg per day CPT as single-agent therapy for patients with RRMM.

**Methods:**

Patients with RRMM were treated once daily with CPT (2.5 mg/kg, intravenously) for 14 consecutive days for each 21-day cycle. Clinical response and toxicity were assessed after each treatment cycle.

**Results:**

Twenty-seven patients received CPT. Using the European Group for Blood and Marrow Transplantation criteria, we calculated the overall response rate of 33.3% with 1 near-complete response (nCR) and 8 partial responses (PRs). The clinical benefit rate (48.1%) included 1 nCR, 8 PRs, and 4 minimal responses. The most common treatment-related adverse events (TRAEs) were fever, aspartate aminotransferase elevation, alanine aminotransferase elevation, leucopenia, rash, neutropenia, and thrombocytopenia. We graded toxicity using the Common Toxicity Criteria for Adverse Events, version 3.0, and determined that 37.0% of patients had at least 1 grade 3–4 TRAE.

**Conclusions:**

CPT as a single agent can elicit a response in patients with RRMM and is well tolerated. Further clinical investigation is warranted.

*Trial Registration* ChiCTR-ONC-12002065 http://www.chictr.org/cn

## Background

Multiple myeloma (MM) is a malignancy of mature plasma cells. In the United States, it is the second most common hematologic malignancy. Recently, new treatments, including autologous stem cell transplantation, proteasome inhibitors (e.g., bortezomib), and immunomodulatory drugs (IMiDs; e.g., thalidomide and lenalidomide), have substantially improved the response and survival rates of patients with MM [1]. However, relapse and drug resistance remain inevitable. Therefore, additional novel and more effective treatments are urgently needed for patients with relapsed or refractory MM (RRMM).

In 1995, apoptosis ligand 2/tumor necrosis factor-related apoptosis-inducing ligand (Apo2L/TRAIL) was discovered as a new member of the tumor necrosis factor superfamily [2]. It binds to death receptor 4 (DR4) and death receptor 5 (DR5) on the cell surface to form the death-inducing signaling complex, thereby activating the caspase cascade to induce apoptosis, a signaling event known as the extrinsic apoptotic pathway [3]. Apo2L/TRAIL selectively and quickly induces cell apoptosis in many solid tumors and hematologic malignancies, while exhibiting no toxicity to normal cells [4, 5].

Circularly permuted TRAIL (CPT) is a novel mutant form of recombinant human Apo2L/TRAIL that is currently in clinical development for the treatment of MM and other hematologic malignancies. The primary molecular feature of CPT is amino acid (aa) 121–135 at the N-terminus of wild-type Apo2L/TRAIL, which is connected to aa 135–281 at its C-terminus via a flexible linker. Consequently, compared with wild-type Apo2L/TRAIL, CPT has better stability, demonstrates more potent anti-tumor activity, and has a slightly longer in vivo half-life in mice, rats, and humans [6].

For pretreated patients with either hematologic malignancies (including lymphoma and MM) or advanced solid tumors (e.g., lung, colorectal, and renal cancers), a phase I study (unpublished data) evaluated the safety of CPT monotherapy by single-dose escalation (0.5–3.5 mg/kg) and by multiple-dose escalation (1.0–2.5 mg/kg). In the multiple-dose escalation, CPT was administered once daily for 14 consecutive days for each 21-day cycle. Unexpectedly, 2 of 3 patients with RRMM achieved a partial response after the first cycle of CPT treatment at the 2.5-mg/kg dose. This early evidence of activity of single-agent CPT in patients with RRMM caused the dose-escalation study to be terminated, while the maximum tolerated dose (MTD) was not reached. MTD was defined as the highest dose level at which 33% or fewer patients experienced a dose-limiting toxicity (DLT), according to the 3+3 design. DLT was predefined as any of the following that occurred during the first course of treatment and was determined to be possibly, probably, or definitely related to study treatment: (a) grade 3 or higher non-hematologic toxicities and (b) grade 4 hematologic toxicity. In this phase I trial of 28 patients, the most common treatment-related adverse events (TRAEs) were alanine aminotransferase (ALT) elevation (42.9%),

aspartate aminotransferase (AST) elevation (32.1%), fever (14.3%), fatigue (10.7%), nausea (10.7%), and vomiting (10.7%). Of these TRAEs, 14.3% were grade 3 or 4, all were associated with hepatotoxicity, and all were resolved by treating the symptoms or discontinuing CPT treatment (unpublished data).

Preliminary results suggested that single-agent CPT, given at a well-tolerated dose of 2.5 mg/kg, once daily for 14 consecutive days for each 21-day cycle, has promising anti-tumor activity in patients with RRMM. Accordingly, this dose and schedule was then chosen as the recommended dose for the current phase II trial that targets a cohort of patients with RRMM only. Therefore, our phase II, single-arm, open-label, multicenter clinical trial further evaluated the safety and efficacy of single-agent CPT, given at 2.5 mg/kg once daily for 14 days for each 21-day cycle, in patients with RRMM.

## Patients and methods

### Ethics

The study was approved by the Institutional Review Boards of all participating institutions, including Beijing Chao-Yang Hospital (Capital Medical University), Institute of Hematology and Blood Diseases Hospital (Chinese Academy of Medical Science & Peking Union Medical College), Shanghai Changzheng Hospital (the Second Military Medical University), and the Second Hospital of Hebei Medical University. Informed consent was obtained from each patient. The clinical trial registration id is ChiCTR-ONC-12002065.

### Patient eligibility

To be eligible for the study, patients with measurable MM must have received at least one prior treatment before relapse or becoming refractory to the prior treatment. Additional inclusion criteria included (a) at least 18 years of age; (b) a white blood cell count of 2.0 × 10^9^/L or higher, a neutrophil count of 1.0 × 10^9^/L or higher, a platelet count of 30 × 10^9^/L or higher, and a hemoglobin count of 60 g/L or higher; (c) total serum bilirubin, ALT, and AST levels each 1.25 times or below the upper limits of normal; (d) a Karnofsky Performance Status score of 60 or higher; and (e) an expected survival time of 3 months or longer.

Exclusion criteria included pregnancy; lactation; history of allergic reactions to proteins or other biological products; an allergic constitution; previous history of viral hepatitis, cirrhosis, alcoholic liver disease, or drug-induced hepatitis (based on the observation that hepatotoxicity was commonly observed in the phase I trial of single-agent CPT); congestive heart failure (New York Heart Association Functional Class III–IV); symptomatic ischemia; conduction abnormalities uncontrolled by conventional intervention; myocardial infarction within 6 months of first dose; other tumors; or deemed otherwise unsuitable by the investigators.

### Study design and treatment

For our study, we used the standard single-stage design [7]. This design requires at least 27 patients to test the null hypothesis that the true overall response rate (ORR) for CPT treatment is at most 5% versus the alternative hypothesis that the largest true ORR is 20% or more with a one-sided α of 0.05 and power (1 − β) of 0.8.

CPT (5 mg/vial) was provided as a lyophilized preparation. The drug was dissolved in 250 mL of 5% glucose. It was then administered at a dose of 2.5 mg/kg via continuous intravenous infusion over 90 ± 15 min once daily for 14 consecutive days for each 21-day cycle, for a total of two cycles.

### Assessments and endpoints

We assessed therapeutic response at the end of each treatment cycle. Serum levels of the involved immunoglobulins were determined by nephelometry, and urine levels of M-protein were determined by the 24-hour urine tests that measure kappa and lambda light chain. Therapeutic responses were defined as complete response (CR), near-complete response (nCR), partial response (PR), minimal response (MR), no change (NC), and progressive disease (PD), according to the European Group for Blood and Marrow Transplantation criteria [8].

Adverse events were monitored throughout the study and recorded for all patients who received at least one dose of CPT. Adverse event data were collected at the four participating institutions from investigator reports and patient self-reports. Adverse events were graded according to the Common Toxicity Criteria for Adverse Events, version 3.0. Other safety assessments included clinical laboratory tests and vital sign observations. Using the indirect enzyme linked immunosorbent assay (ELISA) test, CPT immunogenicity was evaluated by detecting serum anti-CPT antibodies (IgG or IgM).

Study endpoints were therapeutic response rates, including ORR (defined as the percentage of patients who achieved CR, nCR, or PR) and clinical benefit rate (CBR; defined as the percentage of patients who achieved CR, nCR, PR, or MR), and adverse event incidences.

### Statistical analysis

Student’s *t* test was used for comparing measurement data; the Chi square test or Fisher’s exact test was used for comparing enumeration data. All statistical analyses were two-sided. *P* values less than or equal to 0.05 were considered statistically significant. Statistical analyses were performed using SPSS 17.0 software (SPSS Inc., Chicago, IL, USA).

## Results

### Patient characteristics

At four participating institutions in China, 27 patients (9 women and 18 men) were enrolled between september 2007 and october 2008. Patient characteristics are summarized in Table 1. The median age of patients was 56 years. The median time from diagnosis was 21 months. The median number of prior treatments was 3. More than 85% of patients had previously received glucocorticoids (25 patients) or alkylating agents (23 patients), and 14 patients (51.9%) and 21 patients (77.8%) had received prior bortezomib and IMiD (e.g., thalidomide and lenalidomide) therapy, respectively. Using the International Staging System, 74.1% (20 of 27) of patients were diagnosed with stage II/III MM.

**Table 1:** Baseline characteristics of 27 patients with relapsed or refractory multiple myeloma (RRMM)

Characteristic	No. of patients (%)
Age^a^ (years)	56 (36–77)
Sex	
Men	18 (66.7)
Women	9 (33.3)
ISS stage^b^	
I	6 (23.1)
II	14 (53.8)
III	6 (23.1)
Time since diagnosis^a^ (months)	21 (4–60)
Subtype of disease	
IgG	11 (40.7)
IgA	6 (22.2)
IgD	5 (18.5)
Light chain	5 (18.5)
β2-microglobulin^b^	
Level^a^ (mg/L)	3.4 (1.1–16.7)
<3.5 mg/L	14 (53.8)
≥3.5 mg/L	12 (46.2)
Prior therapy	
Number^a^ (cycles)	3 (1–8)
0–3 cycles	14 (51.9)
>3 cycles	13 (48.1)
Prior regimen	
Glucocorticoids	25 (92.6)
Alkylating agents	23 (85.2)
IMiDs (thalidomide or lenalidomide)	21 (77.8)
Vincristine	19 (70.3)
Bortezomib	14 (51.9)
Both bortezomib and IMiDs	9 (33.3)
Autologous stem cell transplantation	5 (18.5)

*ISS* international staging system, *IMiDs* immunomodulatory drugs

^a^These values are presented as median followed by ranges in the parentheses; other values are presented as number of patients followed by percentages in the parentheses

^b^Baseline β2-microglobulin was not determined in one patient; who could not be grouped into any ISS stage

### Efficacy

All 27 patients were evaluated for therapeutic responses to single-agent CPT. As shown in Table 2, the ORR was 33.3% (9 of 27), in which 1 patient achieved an nCR and 8 patients achieved a PR; additionally, 4 patients achieved an MR, resulting in a 48.1% (13 of 27) CBR (nCR + PR + MR). Three (11.1%) patients and 11 (40.7%) patients had NC and PD, respectively.

**Table 2:** Therapeutic responses of 27 RRMM patients to single-agent circularly permuted TRAIL (CPT) treatment

Response^a^	No. of patients (%)
Near-complete response (nCR)	1 (3.7)
Partial response (PR)	8 (29.6)
Minimal response (MR)	4 (14.8)
No change (NC)	3 (11.1)
Progressive disease (PD)	11 (40.7)
ORR (nCR + PR)	9 (33.3)
CBR (nCR + PR + MR)	13 (48.1)

*ORR* overall response rate, *CBR* clinical benefit rate, *TRAIL* tumor necrosis factor-related apoptosis-inducing ligand

^a^Responses were assessed according to the European Group for Blood and Marrow Transplantation criteria

Post hoc analysis was then carried out to compare ORR or CBR between the subgroups divided upon different baseline characteristics. Patients with baseline serum β2-microglobulin levels of 3.5 mg/L or higher (*n* = 12) had an ORR of 50.0% and a CBR of 66.7% that were clearly higher than those for patients with serum β2-microglobulin levels lower than 3.5 mg/L (*n* = 14) (ORR, 14.3%; CBR, 28.6%); the β2-microglobulin level for the remaining one patient was not available. Interestingly, patients who received more than three prior therapies (*n* = 13) had an ORR of 46.2% and a CBR of 61.5% that were higher than those of patients who received three or fewer prior therapies (*n* = 14) (ORR, 21.4%; CBR, 35.7%). Moreover, patients who received prior bortezomib treatment and then became resistant to or intolerant of bortezomib (*n* = 14) had a higher ORR of 42.9% and CBR of 57.1% than patients who were not treated previously with bortezomib (*n* = 13) (ORR, 23.1%; CBR, 38.5%). In addition, the ORR and CBR of patients who had previously received both bortezomib and IMiDs (*n* = 9) were 33.3% and 55.5%, respectively. However, while all of the differences in ORR and CBR between these subgroups of patients with different baseline characteristics were noteworthy, they were not statistically significant (*P* > 0.05 for all comparisons described above). These findings suggest that single-agent CPT is an effective treatment for patients with RRMM, including those patients who have been aggressively treated previously with regimens containing new agents such as bortezomib and IMiDs.

### Safety

All 27 patients received at least one cycle of CPT treatment; of them, 21 (77.8%) completed two cycles of treatment. Treatment was discontinued after the first cycle in only 6 patients; of these discontinuations, 4 were because of adverse events, and 2 were because of disease progression. These results suggest that single-agent CPT is well tolerated in patients with RRMM.

Regardless of whether adverse events were related to CPT treatment, the most common events (defined as incidence in ≥10% of patients) were AST elevation (63.0%), fever (59.3%), ALT elevation (55.6%), leucopenia (48.2%), thrombocytopenia (25.9%), neutropenia (18.5%), upper respiratory infection (18.5%), anemia (14.8%), rash (14.8%), fatigue (14.8%), hypokalemia (11.1%), lactate dehydrogenase (LDH) elevation (11.1%), pneumonia (11.1%), cough (11.1%), pharyngitis (11.1%), abdominal pain (11.1%), and diarrhea (11.1%). As shown in Table 3, the most common TRAEs were fever (48.1%), AST elevation (48.1%), ALT elevation (44.4%), leucopenia (25.9%), rash (14.8%), neutropenia (14.8 %), and thrombocytopenia (11.1%). Of note, most TRAEs were grade 1–2; grade 3–4 TRAEs were reported in 37.0% (10/27) of patients, including AST elevation (18.5%), leucopenia (11.1%), neutropenia (11.1%), ALT elevation (7.4%), thrombocytopenia (7.4%).

**Table 3:** Incidences of treatment-related adverse events^a^ after single-agent CPT treatment in 27 patients with RRMM

Adverse event	All grades^b^	Grade 3/4^c^
Overall adverse events	24 (88.9)	10 (37.0)
Fever	13 (48.1)	0 (0.0)
AST elevation	13 (48.1)	5 (18.5)
ALT elevation	12 (44.4)	2 (7.4)
Leukopenia	7 (25.9)	3 (11.1)
Rash	4 (14.8)	0 (0.0)
Neutropenia	4 (14.8)	3 (11.1)
Thrombocytopenia	3 (11.1)	2 (7.4)
Blood bilirubin elevation	2 (7.4)	1 (3.7)
Creatinine elevation	2 (7.4)	0 (0.0)
Upper respiratory infection	2 (7.4)	0 (0.0)
Uric acid elevation	1 (3.7)^d^	1 (3.7)

Only those toxicities deemed possibly, probably, or definitely related to the treatment are included in the table. Note: a patient may have had more than one adverse event

*AST* aspartate aminotransferase, *ALT* alanine aminotransferase

^a^Adverse events were assessed according to the Common Toxicity Criteria for Adverse Events. Data are presented as number of patients followed by percentage in the parentheses

^b^Adverse event reported in at least 5% of the treated patients

^c^All patients with grade 3 or 4 adverse events

^d^Although uric acid elevation occurred in only 1 patient (<5%), this adverse event was listed because it was grade 3

Serious adverse events were reported in 3 patients (11.1%). Of these, during the second cycle of CPT treatment, 1 patient experienced treatment-related grade 3 ALT elevation and grade 4 AST elevation, which were alleviated and resolved within 1 week of treatment discontinuation, and was given symptomatic treatments such as medications to protect hepatocytes from oxidative and inflammatory damages (e.g., glutathione, polyene phosphatidylcholine). Two patients had either a fever during the second cycle of treatment or a lung infection at the end of the first cycle, both of which were deemed unrelated to CPT treatment and resolved by symptomatic therapies (e.g., antipyretics, antibiotics, antifungal agents), respectively.

Adverse events caused treatment discontinuation in 4 patients. Of these, 2 patients experienced either liver dysfunction or lung infection, as described above. In addition, 1 patient experienced treatment-related grade 3 elevation of AST, ALT, and bilirubin during the second cycle of CPT treatment, and 1 patient experienced treatment-unrelated grade 1 diarrhea and grade 1 fever during the second cycle of treatment.

Finally, 18 patients were evaluated for serum levels of anti-CPT antibodies; IgG and IgM antibodies were detected in 2 (11.1%) and 5 (27.8%) patients, respectively. However, no association was observed between serum anti-CPT level and therapeutic response (either ORR or CBR) or adverse events.

## Discussion

In this phase II study, single-agent CPT at a well tolerated dose (2.5 mg/kg per day) exhibited a response in patients with RRMM. In the past decade, novel treatments for patients newly diagnosed with MM have substantially improved outcomes; however, nearly all patients who respond to initial therapies eventually relapse and become refractory to current treatments, including new drugs [1]. The prognosis of these patients with RRMM remains dismal. Therefore, new and more effective therapies are urgently needed. In this phase II trial, CPT as a single-agent therapy showed promising activity: RRMM patients who had been aggressively treated previously (median, three prior therapies) had a 33.3% ORR and a 48.1% CBR. Interestingly, CPT was also effective for RRMM patients who had received prior regimens containing new anti-MM agents, including bortezomib (ORR, 51.9%) and bortezomib plus IMiDs (ORR, 33.3%). Thus, the present study showed activity of single-agent CPT in the treatment of MM.

In preclinical settings, pro-apoptotic receptor agonists (PARAs) have shown potent and selective anti-tumor activity; however, few clinical studies of PARAs have reported activity in the treatment of patients with cancer, including MM. For example, Ashkenazi and Herbst [9] summarized that rhApo2L/TRAIL and other PARAs had synergistic effects when combined with cytotoxic agents, including irinotecan, camptothecin, 5-fluorouracil, carboplatin, paclitaxel, doxorubicin, and gemcitabine. Additionally, the combination of Apo2L/TRAIL and bortezomib led to enhanced activity in the induction of apoptosis in cell lines of a variety of solid tumors [10–12] and hematologic malignancies [13–15]. To this end, bortezomib up-regulates DR4/DR5 expression while reducing cellular FLICE (FADD-like IL-1β-converting enzyme)-inhibitory protein (c-FLIP) levels, thereby effectively overcoming Apo2L/TRAIL resistance in MM cells [16]. However, HGS-ETR1 (mapatumumab, a DR4 agonistic monoclonal antibody [17]), when administrated in combination with bortezomib and when compared with bortezomib alone, has failed to improve outcomes of patients with advanced MM.

In patients with MM, high serum β2-microglobulin levels have been reported to be associated with poor prognosis. In the study by Bergsagel [18], 66.7% of patients with higher β2-microglobulin levels (i.e., ≥3.5 mg/L) achieved an MR or better, whereas patients with lower β2-microglobulin levels (i.e., <3.5 mg/L) had a CBR of only 28.6%. These findings suggested that CPT might benefit MM patients who have higher baseline levels of serum β2-microglobulin, which is a well-established biomarker for poor prognosis of this disease [18]. In general, RRMM patients who have been aggressively pretreated usually respond poorly to current regimens, including the new drugs (e.g., proteasome inhibitors and IMiDs). However, in our study, we observed an interesting trend, namely that, after CPT treatment, both ORR and CBR were notably higher in patients who had received more than three prior treatments than in patients who had received three or fewer prior treatments (ORR, 46.2% vs. 21.4%; CBR, 61.5% vs. 35.7%). We observed similar results when we compared response rates between the subgroups of patients who had been previously treated with bortezomib (ORR, 42.9%; CBR, 57.1%) or bortezomib plus IMiDs (ORR, 33.3%; CBR, 55.5%) and those who had not received prior bortezomib treatment (ORR, 23.1; CBR, 38.5%). One possibility for not observing cross-resistance between CPT and current anti-MM agents is that CPT primarily targets DR4 and DR5 to induce apoptosis through activation of the extrinsic apoptotic pathway, whereas these agents (e.g., bortezomib and IMiDs) act mostly via the mitochondria-mediated intrinsic apoptotic pathway. Moreover, a potential explanation for the likelihood of better responses to CPT in patients who received prior bortezomib is that this agent might modulate the signaling molecules (e.g., DR4/DR5 [18]) related to the extrinsic apoptotic pathway, thereby sensitizing MM cells to CPT that targets this pathway. The differences in the response rates (both ORR and CBR) between those subgroups of patients with different baseline characteristics were not statistically significant, most likely because of the small sample size; thus, further studies are needed to validate these preliminary but interesting findings. It is too soon as well to draw any conclusions about which subsets of RRMM patients would likely benefit more from single-agent CPT treatment.

In this phase II study, in addition to elevated ALT and AST levels and fever, which were most frequently observed in the phase I study, the most common adverse events were leucopenia, neutropenia, thrombocytopenia, and rash. The reason for the common hematologic adverse events is most likely due to RRMM itself, which often causes hematologic abnormalities. While gastrointestinal adverse events and fever were very common in an earlier trial of rhApo2L/TRAIL in patients with solid tumors [19], elevated AST and ALT levels and fever were more common in the present cohort of RRMM patients who received single-agent CPT. One possible reason is that in our phase II study, patients received prolonged treatment with CPT (i.e., 14 days for each cycle), whereas in the phase I trial, patients received rhApo2L/TRAIL for only 5 days for each cycle. In the clinical development of PARAs, liver toxicity has been a major concern [20]. In our study, more than half of patients who received single-agent CPT experienced elevated AST and/or ALT levels. However, it is noteworthy that most of these hepatic adverse events were grade 1 or 2, and nearly all were manageable with symptomatic treatments and/or discontinuation of CPT. Interestingly, most patients with elevated AST levels had normal ALT levels, but this was often accompanied by elevated LDH levels. This phenomenon was usually observed during the first cycle of CPT treatment and very rarely afterwards. AST levels returned to normal or near-normal within 1 week; LDH levels declined more slowly, to normal or near-normal within 2 weeks. Interestingly, patients with transient elevation of AST levels and/or LDH levels but normal ALT levels were more likely to achieve an MR or better, suggesting that AST elevation with normal ALT levels might not be associated with liver toxicity but rather tumor lysis in response to CPT treatment [21]. In contrast to transient AST elevation that peaked at day 2 or day 3 of the first cycle and then declined quickly, ALT elevation was more sustained during the CPT dosing period but declined and eventually recovered after discontinuation of CPT. Most patients (80.0%) had grades 1–2 ALT elevation, with normal bilirubin level. Only two patients discontinued CPT treatment because of liver toxicity; of these, one patient had grade 4 AST elevation and grade 3 ALT elevation, which was reported as a serious adverse event, and another patient had grade 3 elevation of AST, ALT, and bilirubin, both of which occurred during the second cycle of CPT treatment. Of note, in both cases, these liver adverse events resolved after discontinuation of CPT. Therefore, although patients in our study commonly experienced elevated AST and ALT levels, the elevated levels were mostly mild or moderate and all manageable. Together, these results suggest that single-agent CPT is well tolerated by patients with RRMM.

Our study does, however, have limitations. First, therapeutic response (i.e., ORR and CBR), rather than the gold standard endpoint overall survival, was selected as the primary outcome to evaluate efficacy of single-agent CPT in patients with RRMM. Second, CPT was administered for a relatively short period of time (only two 21-day cycles). Third, the follow-up time, which ended after the second cycle of CPT treatment, was also short. Collectively, these limitations prevent this study from providing more definitive evidence for the efficacy of single-agent CPT on RRMM. For example, whether longer treatment (e.g., increasing the number of intervention cycles) with single-agent CPT would be more beneficial while not increasing toxicity is still unknown. More importantly, whether single-agent CPT would improve long-term outcomes (e.g., progression-free survival, duration of response, or overall survival) of patients with RRMM is also still unknown.

## Conclusions

This phase II study provides the first evidence supporting the promising therapeutic activity of single-agent CPT in the treatment of RRMM patients, including those who were aggressively treated previously with current regimens containing novel agents, such as bortezomib or IMiDs. It also demonstrated that CPT at the dose of 2.5 mg/kg, given once daily for 14 days per cycle for two cycles, was well tolerated. Although mild and moderate hepatic adverse events were relatively common, nearly all were manageable. Therefore, the single-agent CPT therapy and the regimens that combine CPT with first-line anti-MM agents (e.g., bortezomib and IMiDs) warrant further clinical investigation. Accordingly, a phase III trial to further develop CPT as a single-agent therapy and as part of combination therapies for RRMM is currently ongoing.

## Abbreviations

Apo2L/TRAIL: apoptosis ligand 2/tumor necrosis factor-related apoptosis-inducing ligand
CBR: clinical benefit rate
CPT: circularly permuted TRAIL
CR: complete response
DR4/DR5: death receptor 4/5
IMiDs: immunomodulatory drugs
MM: multiple myeloma
MR: minimal response
NC: no change
nCR: near-complete response
ORR: overall response rate
PARAs: pro-apoptotic receptor agonists
PD: progressive disease
PR: partial response
RRMM: relapsed or refractory multiple myeloma
TRAE: treatment-related adverse event
